# Intervening on the Developmental Course of Children With Borderline Intellectual Functioning With a Multimodal Intervention: Results From a Randomized Controlled Trial

**DOI:** 10.3389/fpsyg.2020.00679

**Published:** 2020-04-21

**Authors:** Valeria Blasi, Michela Zanette, Gisella Baglio, Alice Giangiacomo, Sonia Di Tella, Maria Paola Canevini, Mauro Walder, Mario Clerici, Francesca Baglio, the BIF Group, D. Belotti

**Affiliations:** IRCCS, Fondazione don Carlo Gnocchi, Milan, Italy; ASST S. Paolo and S. Carlo Hospital, Milan, Italy; IRCCS, Fondazione don Carlo Gnocchi, Milan, Italy and University of Milan, Milan, Italy; ^1^IRCCS Fondazione Don Carlo Gnocchi, Milan, Italy; ^2^Department of Health Sciences, University of Milan, Milan, Italy; ^3^ASST S. Paolo and S. Carlo Hospital, Milan, Italy; ^4^Department of Pathophysiology and Transplantation, University of Milan, Milan, Italy

**Keywords:** adverse social environment, school failure, intellectual functioning, behavioral competences, emotion regulation, child psychiatry, multimodal rehabilitation

## Abstract

An adverse social environment is a major risk factor for borderline intellectual functioning (BIF), a condition characterized by an intelligence quotient (IQ) within the low range of normality (70–85) with difficulties in the academic achievements and adaptive behavior. Children with BIF show impairments in planning, language, movement, emotion regulation, and social abilities. Moreover, the BIF condition exposes children to an increased risk of school failures and the development of mental health problems, and poverty in adulthood. Thus, an early and effective intervention capable of improving the neurodevelopmental trajectory of children with BIF is of great relevance.

**Aim:**

The present work aims to report the results of a randomized controlled trial (RCT) in which an intensive, integrated and innovative intervention, the movement cognition and narration of the emotions (MCNT) was compared to standard speech therapy (SST) for the treatment of children with BIF.

**Methods:**

This was a multicenter, interventional, single blind RCT with two groups of children with BIF: the experimental treatment (MCNT) and the treatment as usual (SST). A mixed factorial ANOVA was carried out to assess differences in the effectiveness between treatments. Primary outcome measures were: WISC III, Child Behavior Checklist (CBCL), Vineland II, and Movement ABC.

**Results:**

MCNT proved to be more effective than SST in the increment of full-scale IQ (*p* = 0.0220), performance IQ (*p* < 0.0150), socialization abilities (*p* = 0.0220), and behavior (*p* = 0.0016). No improvement was observed in motor abilities. Both treatments were linked to improvements in verbal memory, selective attention, planning, and language comprehension. Finally, children in the SST group showed a significant worsening in their behavior.

**Conclusion:**

Our data show that an intensive and multimodal treatment is more effective than a single domain treatment for improving intellectual, adaptive and behavioral functioning in children with BIF. These improvements are relevant as they might represent protective factors against the risk of school failure, poverty and psychopathology to which children with BIF are exposed in the adult age. Limitations of the study are represented by the small number of subjects and the lack of a no-treatment group.

**Clinical Trial Registration:**

ISRCTN Registry (isrctn.com), identifier ISRCTN81710297.

## Introduction

Several factors related to the social environment such as low socio-economic status, maltreatment, and high levels of maternal stress represent the major causes for borderline intellectual functioning (BIF) (Bradley and Corwyn, 2002; Marcus Jenkins et al., 2013; Peltopuro et al., 2014; Hassiotis et al., 2019). BIF is a condition characterized by a mental functioning at the border between normal intellectual functioning and intellectual disability, which means an IQ within 1 and 2 standard deviations below the mean of the normal curve of the distribution of intelligence with an impact on adaptive abilities (Salvador-Carulla et al., 2013; Wieland and Zitman, 2016). In primary school age, children with BIF present major difficulties in school achievements due to learning difficulties in more than one domain, difficulties in executive functions, such as attention, concentration, planning, and inhibition of impulsive responses, in memory, and motor skill limitations (Alloway, 2010; Vuijk et al., 2010; Salvador-Carulla et al., 2013; Pulina et al., 2019). Furthermore, limitations in social skills, emotional competencies and behavioral problems affect social participation of these children (Kavale and Forness, 1996; Baglio et al., 2016). Children with BIF are thus at high risk of school failures and dropout (Karande et al., 2008; Shaw, 2008, 2010), and to develop psychiatric problems in the adult age (Chaplin et al., 2006; Douma et al., 2007; Ali and Hassiotis, 2008; Gigi et al., 2014; Hassiotis, 2015; Hassiotis et al., 2019). Recent studies established a prevalence of BIF ranging from 7 to 12% (Salvador-Carulla et al., 2013; Hassiotis, 2015).

Although intelligence is one of the most heritable behavioral traits, its heritability seems to account for about 20% to 40% in infancy (Plomin and Deary, 2015). Intelligence, indeed, appears to be stable during adolescence to adulthood, but childhood environment can play a crucial role, especially in families with low socio-economic status (SES). The complex interplay between genes and environment during development is supported by findings from a longitudinal study that followed a large cohort of 14,853 children (von Stumm and Plomin, 2015). Results showed that 2-year-old children from low SES environments had an average of six points lower IQ compared to their high SES peers; by the age of 16, this gap had nearly tripled.

The link between BIF and social environment is likely related to the interplay between adverse life conditions and brain development. Childhood is indeed a critical period because of the dramatic changes that occur in the brain. It has been demonstrated that low SES correlates with both reduced learning abilities and abnormal brain development in several critical regions including the hippocampus, amygdala, parahippocampal and sensory-motor cortices, and limbic system connectivity (Hanson et al., 2011; Baglio et al., 2014; Hair et al., 2015; Blasi et al., 2019). These data are relevant as they indicate that children with BIF might be at risk of learning difficulties and emotional problems at a very early age. All the aforementioned findings highlight the necessity of an early and effective intervention capable of improving the clinical and neurodevelopmental course of children with BIF by exploiting the substantial plasticity of the developing brain (Johnston, 2009).

No specific rehabilitation approach and guidelines are available at the moment for children with BIF. The usual care provided by the national health system in Italy is focused on the learning difficulties and consists of standard speech therapy (SST). Furthermore, in the mainstream Italian school system, children with BIF are classified as Special Educational Needs (SEN). Children with SEN have a personalized and simplified school program (PSSP) whose purpose is to warrant compensatory tools and dispensatory measures (i.e., the prescription to use facilitation devices such as a calculator and/or a computer) as well as to simplify the educational approach. Both PSSP and SST, though, are focused only on academic abilities without considering the complexity and multiplicity of the difficulties and needs of this population.

As specific interventions for children with BIF are lacking, we developed a multimodal treatment (Blasi et al., 2017a) based on three main theoretical considerations. First, intelligence seems to be a multidimensional and dynamic process that plays a pivotal role in the development of truly adaptive abilities (Gottfredson, 1997). Intelligence is one of the best “predictors of important life outcomes such as education, occupation, mental and physical health and illness, and mortality” (Plomin and Deary, 2015). For this reason a treatment that is effective in the increment of the IQ can represent a protective factor from social disadvantage in adulthood. Second, the development of emotional, cognitive and motor skills is highly correlated in both typical development (Wassenberg et al., 2005; Inkster et al., 2016) and in children with BIF (Hartman et al., 2010; Houwen et al., 2016). Therefore, effective rehabilitation interventions during childhood should include all these domains. Finally, higher levels of education and living in cognitively stimulating environments result in greater cognitive reserve that can positively impact neurodevelopment (Schapiro and Vukovich, 1970).

Based on these considerations, we designed a treatment named movement, cognition and narration of the emotions treatment (MCNT) (Blasi et al., 2017a). Central aspects of MCNT are the intensity and the integration of the approach. Children attend the program for a whole school year (9 months), 3 h per day, Monday through Friday. MCNT operates through a highly enriched and motivating approach in which children are divided into three “teams” that, in rotation, attend three laboratories, one for each domain: cognition, movement, and emotions. The MCNT program is integrated with the school programs and with the families through the engagement of teachers and parents in the finality and the strategies used in the program (Blasi et al., 2017a).

The aim of the present work is to report the results of the previously published Study Protocol (Blasi et al., 2017a), in which a detailed description of all the procedures and treatment adopted is available. The aim of the trial was to investigate the efficacy of the MCNT intervention in the recovery of the BIF condition and to compare it with SST in promoting complex reasoning, motor, behavioral and adaptive skills.

## Materials and Methods

### Study Design and Participants

**FIGURE 1:**
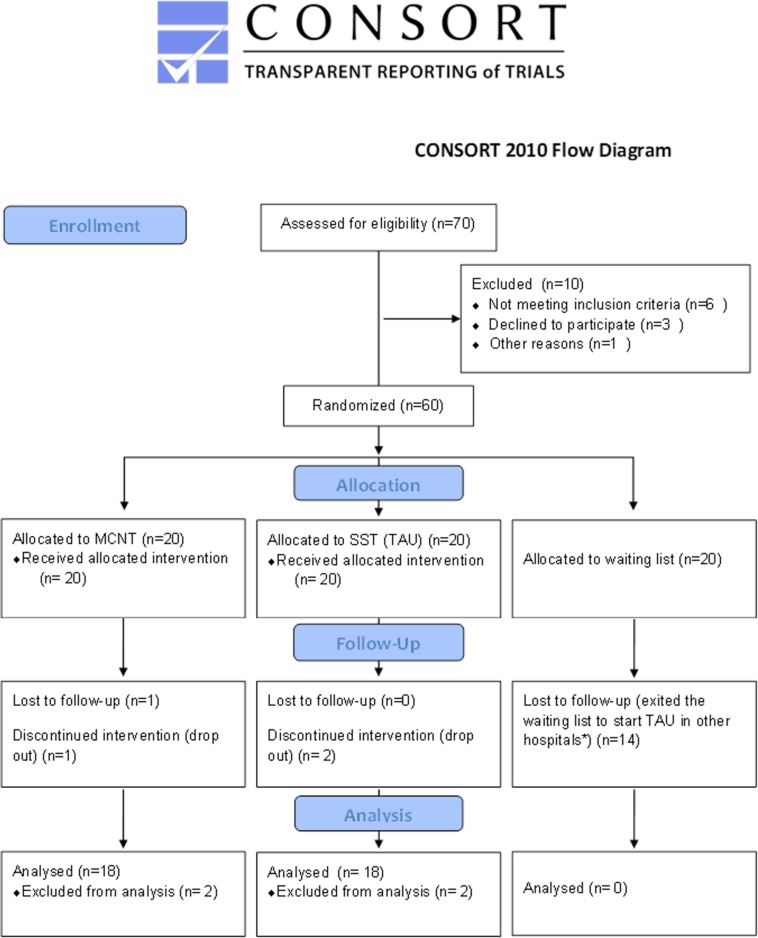
CONSORT flow diagram of the RCT. *According to the Ethics’ Committee recommendations, subjects in the no-treatment group could not be kept on the waiting list and deprived of the conventional treatment when the same treatment became available. Consequently, 14 children belonging to this group exited the study before the follow-up assessment. MCNT, Movement Cognition and Narration of the emotions Treatment; SST, standard speech therapy; TAU, treatment as usual.

This was a multicenter, interventional, single blind, randomized controlled study (RCT) originally designed with three groups of children with BIF: group 1- children treated with SST (treatment as usual, *N* = 20); group 2- children treated with MCNT (experimental treatment, *N* = 20); group 3- children on the waiting list for SST (no treatment; *N* = 20) (Figure 1).

The study was approved by the Ethics Committee of the Don Gnocchi Foundation (DGF) and of the ASST S. Paolo and S. Carlo Hospital. All parents signed a written informed consent at the first meeting.

Seventy children were recruited from the Child and Adolescent Neuropsychiatry Unit of the two Medical Centers involved (DGF, and ASST S. Paolo and S. Carlo Hospital) where they were referred to for their difficulties in terms of school achievements and/or socialization. All children were allocated, evaluated and treated at DGF (Figure 1). Ten children were excluded because they did not meet the inclusion criteria described below and/or declined to participate. Moreover, according to the Ethics’ Committee recommendations, subjects in the no-treatment group could not be kept on the waiting list and deprived of the conventional treatment when the same treatment became available. Due to unexpected opening of new opportunities of treatment outside our Institution, 14 children belonging to this group exited the study before the T1 assessment. For this reason, the final sample included forty children belonging to the two treatments groups (Figure 1).

The measures of primary and secondary outcome were determined at two time points; within 2 months prior to the beginning of the treatment (T0) and within 2 months after the end of the treatment (T1). Two psychologists, blinded to the intervention received, evaluated children before and after treatment. Two outcomes, the Child Behavioral Checklist (CBCL 6-18) (Achenbach and Rescorla, 2001, 2007; Achenbach, 2011) and the Vineland II (Sparrow et al., 2005), were not blind to the experimental condition because the questionnaire was completed by the parents. Following two drop-outs in each group, 18 children completed MCNT and 18 completed SST.

The inclusion criteria were: age range between 6 to 11 years old and attending primary mainstream school; with a Full Scale Intelligence Quotient (FSIQ) score ranging from 70 to 85 (±5) determined with the Wechsler Intelligence Scale for Children-III (WISC-III) (Wechsler, 2006); presence of learning disabilities assessed with the standardized test battery for developmental dyslexia and dysorthographia (DDE-2) (Sartori et al., 2007) and dyscalculia (AC-MT 6-11) (Cornoldi et al., 2012); presence of an impact on daily life of the above mentioned difficulties as measured by the Child Behavioral Checklist (CBCL 6-18) (Achenbach and Rescorla, 2001; Achenbach and Rescorla, 2007; Achenbach, 2011).

Exclusion criteria were: presence of major neuropsychiatric disorders (such as ADHD and autism spectrum disorder); presence of neurological conditions such as epilepsy, traumatic brain injury, brain malformation and infectious disease involving the central nervous system. Other exclusion criteria considered were: the presence of systemic diseases such as diabetes or dysimmune disorders, genetic syndromes such as Down syndrome or Fragile X syndrome. Furthermore, a positive history for psychoactive drugs, particularly referring to current or past use of psychostimulants, neuroleptics, antidepressants, benzodiazepines and antiepileptic drugs were also considered exclusion criteria.

### Randomization and Blinding

Randomization occurred after screening and baseline assessment (T0). Subjects were randomly assigned to the groups. The randomization process was performed using a computer algorithm^1^ by an independent operator not involved in the study. The evaluation in both pre- and post-treatment was conducted by two psychologists blind to group allocation.

### Sample Size and Statistical Analysis

Due to four drop-outs (two children for each group), the final sample was represented by 36 children: mean age was 8.23 (sd 1.46) for MCNT group (M/F = 8/10) and 8.22 (sd 1.26) for SST group (M/F = 10/8).

Due to the drop-out of the waiting list group and the consequent change in the study design, we performed a new *a priori* power calculation. We calculated the effect size on preliminary data from a separate sample of 45 children treated with MCNT and 47 with SST for the primary outcome measure (FSIQ) using G*Power version 3.0.10. Results showed a mean difference value between groups after treatment of eight points, with a standard deviation of 10, and a correlation value among repeated measure of 0.3. For a given expected power of 0.82 and an effect size of 0.41, the estimated sample size was 36. Considering a 10% drop-out rate, the number of subjects required was 40.

Statistical analysis on outcome measures was conducted using SPSS Statistics 24. All variables were tested for skewness and kurtosis to check for normality. An independent samples *t*-test assessed baseline differences between groups for demographic and IQ data.

A mixed factorial ANOVA, with type of intervention (MCNT and SST) as the independent variable and outcome measures (IQ, M-ABC; Vineland II, CBCL, and neuropsychological data) as the repeated measures, was carried out to assess the main effect of treatment (Time T0 vs. T1) and differences in effectiveness between treatments (Time by Group interaction). *Post hoc* comparisons were carried out to test for simple main effects. Due to the small number of subjects included in the study and to avoid missing a possible effect, we applied a false discovery rate (FDR) correction according to Benjamini and Yekutieli (2001) to account for multiple comparisons. Moreover, due to the small number of subjects we did not perform an intention to treat analysis for the missing data.

### Interventions

In our study, two types of interventions were carried out: MCNT, which represents the experimental intervention; and SST, the treatment as usual. In Italy, SST is the only treatment offered by the National Health System for children with BIF, with the aim to improve their difficulties in learning and verbal comprehension. Both treatments were carried out at DGF in a hospital setting, and lasted for 9 months and there were regular meetings between the professionals, the families and teachers of the children. Both treatments were also discussed during regular weekly meetings among professionals. MCNT was based on a multidimensional approach and children worked in small groups while SST was focused on learning abilities and children worked one-to-one with the speech therapist.

For a comprehensive description of both rehabilitative approaches, see the Study Protocol (Blasi et al., 2017a).

#### The Movement Cognition and Narration of the Emotions Treatment (MCNT)

Children worked in small groups (five to six children each), for 3 h each day, 5 days a week, Monday to Friday, for 9 months. To encourage cooperative learning within each group and to promote a degree of competition between groups, children were divided into three “teams” named Red, Blue, and Green, for the whole duration of the intervention, according to their global functioning, grade and/or special educational needs. The treatment consisted of: (1) A **Movement Lab**, to improve motor planning and fine and gross motor abilities with a Game Therapy approach using the Wii and Xbox video game platforms; (2) **A Cognitive Lab,** for the empowerment of the executive functions such as working memory, planning abilities, problem solving, and reasoning and language comprehension with the use of the multimedia interactive whiteboard (MIW); (3) an **Emotion Lab,** to learn how to narrate the emotions to help the child to cope with the experiences of her/his daily life.

The Movement Lab involved exercises aimed at improving balance, fine and gross motor abilities, hand–eye coordination to make their movements more fluid, economical, quicker and functional, but also impulsive motor response inhibition, planning, and praxic abilities as well as attention. For instance, the child used the Wiimote to point to a moving target to train attention and higher visual-motor integration, or played Wii Sports with the Wiimote and the Wii Balance Board to train balance and coordination of both upper and lower limbs. Moreover, Wii Music and Wii Party games were used to train rhythm, timing of movement and inhibition of impulsive motor behavior. During the whole process, advanced executive functioning, such as planning competence, working memory and inhibitory control were involved.

The **Cognitive Lab** aimed at promoting language comprehension and expression, executive functions, such as deductive and inductive reasoning, working memory, planning and problem solving, attention and concentration, inhibition of impulsive verbal response. Moreover, children were encouraged to view each problem assuming multiple perspectives, examining possible alternatives, monitoring the decisional processes and promoting links among knowledge with explicit metacognitive strategies. For instance, to promote working memory the neuropsychologist used concrete daily tasks such as thinking of all the sequential acts that need to be prepared when preparing for an activity such as painting. Targeted cognitive stimulation was avoided for two reasons: (1) to avoid introducing a bias in the evaluation of the outcome by using tasks that could resemble those used in the assessment; and (2) to promote metacognitive strategies that are more easily fixed in the long term semantic as well as autobiographical memory and that can generalize to different contexts (Baddeley, 2013). Active participation in the activities was promoted through a cooperative learning approach in which children helped each other and were all responsible for the achievements of the group. Throughout the training, the neuropsychologist referred explicitly to the importance of effort and practice in the increment of their abilities and that intelligence is not a fixed entity but a malleable quality (Blackwell et al., 2007).

The **Emotion Lab** concerned emotions and social skills. The objective was to help children to express, recognize and cope with their own emotions (Blasi et al., 2017b). The underlying idea stems from the psychoanalytic model of Bion (1962) in which the comprehension of the emotional experience is central to the development of thought and to learning. The therapist, a psychologist with a psychotherapy degree, used different approaches to promote the narration of the emotions: symbolic play, reading, inventing and/or dramatizing a story, drawing and talking.

#### Treatment as Usual: Standard Speech Therapy (SST)

Standard speech therapy consisted of individual sessions of 45 min each twice a week for 9 months. The focus was on the training of the academic abilities compromised in the child as assessed by the evaluation at T0 (pre-treatment). To empower these skills, SST used both pencil/paper tools and specific rehabilitation software^2^. In the event of dyslexia or dysorthographia, the main objectives of SST were to increase processing information speed and transcoding, reduce spelling mistakes and expand personal vocabulary and text comprehension. For dyscalculia, images were used to aid reasoning and solving problems such as in the analogical method (Bortolato, 2014; Mehrnoosh and Fusi, 2016).

The empowerment of transversal competences such as phonological competences, verbal comprehension, perception, visual-spatial ability, attention, memory and executive functions were also considered together with the use of compensative tools.

### Assessment Design and Outcomes Measures

All children were evaluated at two time points, within 2 months prior the beginning of the treatment (T0) and within 2 months after the end of the treatment (T1).

Primary outcome measures were: 1. WISC-III (Wechsler, 2006) to measure intellectual functioning and evaluate cognitive profile in light of Verbal and Performance QI; 2. The Movement Assessment Battery for Children (M-ABC) (Henderson and Sugden, 1992), for the assessment of the motor skills. The test provides four scores for manual dexterity, ball skills, static -dynamic balance, and total score; 3. The CBCL 6-18 (Achenbach and Rescorla, 2001, 2007; Achenbach, 2011) to evaluate a child’s adaptive behavior and functioning as seen by the parents. The main scoring for the CBCL is based on eight syndrome scales from DSM5, grouped into two “broad band” scales, Internalizing problems and Externalizing problems along with a Total problems score. The standard scores are scaled so that 50 is average for the youth’s age and gender, with a standard deviation of 10 points. Higher scores indicate greater problems; 4. The Emotional Quotient Inventory-Youth Version (Bar-On and Parker, 2000) was used at T0 for the evaluation of the emotional competencies. Data relative to this test though were not considered interpretable due to the difficulty encountered by children in the comprehension of the items. For this reason, we did not include the test in the post-treatment evaluation since no statistical comparison between T0 and T1 could be performed; the Socialization Scale of the Vineland II (Sparrow et al., 2005) was administered to assess social adaptive abilities.

Secondary outcome measure included: the Modified Bells Test (MBT) (Biancardi and Stoppa, 1997), a barrage test to assess visual scanning efficiency, and visual selective attention; the Tower of London (TOL) to evaluate executive functions and specifically planning ability, strategy decision making and problem solving (Shallice, 1982; Fancello et al., 2006); from the Neuropsychological Evaluation Battery for developmental age 5-11 (BVN 5-11), the Speech Fluency tests using both phonological and semantic keys for verbal executive functions, the Selective Word Retrieval tests, for short and long term verbal memory, the Corsi test for visual spatial short term memory (Bisiacchi et al., 2005); and the Test of Reception of Grammar-2 (TROG2) to evaluate the comprehension capacity of syntactically complex sentences (Bishop, 2003; Suraniti et al., 2009). The scores from all tests are calculated as *Z*-scores with the exception of the TROG2 that is indicated in standard score.

## Results

Table 1 shows the comparison at baseline between the two groups relative to age, SES, IQ, motor abilities, adaptive skills, and behavior. No significant differences between the two groups were detected for age, SES, IQ at baseline, motor abilities and behavior. The Socialization Scale of the Vineland II in which children belonging to the MCNT group had significantly lower scores (*p* = 0.002).

**TABLE 1:** Demographic and IQ data at baseline.

	**SST**	**MCNT**	**MCNT vs. SST *p*-value**

N subjects	18	18	ns
M/F	10/8	8/10	ns

	**Mean (SD)**	**Mean (SD)**	
Age (Years)	8.22 (1.26)	8.23 (1.46)	ns
SES	26.78 (9.61)	24.03 (11.64)	ns
VIQ	77.53 (8.95)	74.12 (9.58)	ns
PIQ	85.12 (8.40)	81.18 (13.50)	ns
FSIQ	78.61 (7.17)	75.11 (8.52)	ns
M-ABC	3.92 (4.03)	6.58 (10.58)	ns
Vineland II*	101.38 (7.96)	86.27 (8.28)	0.002
CBCL (total score)	53.00 (11.51)	64.87 (13.06)	ns

MCNT, Movement Cognition and Narration of the emotions Treatment; SST, Standard Speech Treatment; SES, Socio-Economic Status; VIQ, Verbal IQ; PIQ, Performance IQ; FSIQ, Full Scale IQ; M-ABC, Movement Assessment Battery for Children (data expressed in percentiles); *, Socialization Scale; CBCL, Child Behavior Check List; SD, standard deviation; ns, not significant.

We then proceeded with the factorial ANOVA to assess changes in the primary outcome measures throughout the study (Tables 2, 3). Overall, a significant time by group interaction for the Full scale IQ (*p* < 0.022) and the Performance IQ (*p* < 0.015) was observed, with significant *post hoc* pairwise comparison for the MCNT group only (*p* < 0.001 in both cases). Moreover, the M-ABC evaluation did not show any significant effect in either group, while the Socialization Scale of the Vineland II showed significant time by group interaction (*p* = 0.022) with post-treatment improvement in the MCNT group (*p* = 0.02). Finally, the factorial ANOVA comparing the effect of treatments on the CBCL scores (Table 3) demonstrated significant time by group interaction for all CBCL scores: the Internalizing (*p* = 0.0016), and Externalizing problems scales (*p* = 0.0027), and the Total score (*p* = 0.0016). The pairwise *post hoc* analyses revealed a significant decrease (improvement) in the scores for the MCNT group (*p* = 0.01; *p* = 0.01; *p* = 0.00 for internalizing, externalizing and total score respectively), while the SST group had significant increment (worsening) of the scores (*p* = 0.01; *p* = 0.03; *p* = 0.04).

**TABLE 2:** Results of the ANOVA analysis on the primary outcome measures WISC-III, Movement ABC and Vineland II.

	**SST *N* = 18**	**MCNT *N* = 18**	**Time (T1 vs. T0)**		Time*Group		**Pairwise comparison T0 vs. T1**	
					
**Variable**	**Mean (SD)**	**Mean (SD)**	**F FDR-*p*-value**	**η^2^**	**F FDR-*p*-value**	**η^2^**	**SST *p*-value**	**MCNT *p*-value**
**VIQ**								
T0	77.53 (8.95)	74.12 (9.58)	12.97	0.29	3.90	0.11	ns	**<0.001**
T1	80.29 (9.06)	83.59 (14.84)	**0.0029**		0.0651			
**PIQ**								
T0	85.12 (8.40)	81.18 (13.50)	13.35	0.29	8.14	0.20	ns	**<0.001**
T1	86.47 (12.98)	92.18 (12.03)	**0.0029**		**0.0150**			
**FSIQ**								
T0	78.61 (7.17)	75.11 (8.52)	13.46	0.28	6.36	0.16	ns	**<0.001**
T1	80.56 (11.14)	85.61 (11.83)	**0.0029**		**0.0220**			
**M-ABC**								
T0	3.92 (4.03)	6.58 (10.58)	2.59	0.10	0.69	0.03	ns	0.09
T1	11.77 (14.83)	9.08 (13.94)	0.1942		0.4145			
Vineland II*								
T0	101.38 (7.96)	86.27 (8.28)	6.01	0.21	7.03	0.24	ns	**0.02**
T1	101.00 (10.54)	96.09 (8.53)	**0.0452**		**0.0220**			

MCNT, Movement Cognition and Narration of the emotions Treatment; SST, Standard Speech Treatment; VIQ, Verbal IQ; PIQ, Performance IQ; FSIQ, Full Scale IQ; VCI, Verbal Comprehension Index; POI, Perceptual Organization Index; FDI, Freedom from Distractibility Index; SPI, Speed Processing Index; M-ABC, Movement Assessment Battery for Children (data expressed in percentiles); *, Socialization Scale; SD, standard deviation; η^2^, partial effect size; ns, not significant. Significant results are reported in bold font; FDR, false discovery rate correction.

**TABLE 3:** Results of the ANOVA analysis on the primary outcome measure the CBCL.

	**SST *N* = 12**	**MCNT *N* = 15**	**Time (T1 vs. T0)**		Time*Group		**Pairwise comparison T0 vs. T1**	
					
**CBCL Scale**	**Mean (SD)**	**Mean (SD)**	**F FDR-p value**	**η^2^**	**F FDR-p value**	**η^2^**	**SST**	**MCNT**
**Internalizing**								
T0	52.83(11.52)	64.00(12.44)	0.27	0.01	16.52	0.40	0.01*	**0.01**
T1	62.25(5.01)	56.73(11.96)	0.6914		**0.0016**			
**Externalizing**								
T0	49.08(9.92)	56.00(11.63)	0.03	0.00	13.79	0.36	0.03*	**0.01**
T1	55.33(9.60)	49.13(8.81)	0.8628		**0.0027**			
**Total Score**								
T0	53.00(11.51)	64.87(13.06)	0.98	0.04	17.21	0.41	0.04*	**0.00**
T1	59.92(6.79)	53.60(10.60)	0.4407		**0.0016**			

CBCL, Child Behavior Check List; SST, Standard Speech Treatment; MCNT, Movement Cognition and Narration of the emotions Treatment; SD, standard deviation; η^2^, partial effect size; *, worsening; ns, not significant. Significant results are reported in bold font. FDR, false discovery rate correction.

Table 4 reports data relative to the factorial ANOVA assessing secondary outcome measures derived from the neuropsychological evaluations. The results showed a significant time by group effect only for the Corsi test (visual-spatial memory, *p* = 0.0148) with significant *post hoc* pairwise comparison for the MCNT group (*p* = 0.05). Moreover, for all other variables no significant time by group effect was observed. A significant time effect was observed for short-term (*p* < 0.001) and delayed verbal memory (*p* < 0.001), immediate selective attention (Modified Bells Test rapidity, *p* = 0.0065), planning executive functions (Tower of London, *p* < 0.001), and grammar comprehension (TROG 2, *p* < 0.001), *post hoc* analyses revealed significant effects for both groups. Finally, for the sustained selective attention (Modified Bells Test accuracy) a significant time effect was observed (*p* = 0.0022) with significant *post hoc* analysis only in the SST group (*p*-value < 0.001).

**TABLE 4:** Results of the ANOVA analysis on the neuropsychological assessment, secondary outcome measures.

	**SST *N* = 16**	**MCNT *N* = 18**	**Time (T1 vs. T0)**		Time*Group		**Pairwise comparison T0 vs. T1**	
					
**Variable**	**Mean (SD)**	**Mean (SD)**	**F FDR-p value**	**η^2^**	**F FDR-p value**	**η^2^**	**SST**	**MCNT**
**ST-Verb Memory**								
T0	−2.29(1.75)	−1.56(1.39)	40.36	0.57	3.89	0.11	**<0.001**	**<0.001**
T1	0.13(1,12)	−0.28(1.26)	**<0.001**		0.2606			
**LT-Verb Memory**								
T0	−0.23(0,83)	−0.43(1.21)	22.98	0.43	0.10	0.00	**<0.001**	**<0.001**
T1	0.88 (0.91)	0.55 (1.03)	**<0.001**		0.8780			
**MBT – Rapidity**								
T0	−1.27(0.95)	−1.16(0.68)	9.57	0.25	0.03	0.00	**0.04**	**0.03**
T1	−0.72(0.93)	−0.67(1.07)	**0.0065**		0.8780			
**MBT – Accuracy**								
T0	−2.59(2.16)	−1.92(0.89)	12.79	0.31	2.69	0.08	**<0.001**	Ns
T1	−1.15(1.41)	−1.39(1.77)	**0.0022**		0.3354			
**TOL total score**								
T0	−1.42(0.84)	−0.99(1.02)	15.79	0.35	0.30	0.01	**0.03**	**<0.001**
T1	−0.82(0.94)	−0.20(0.74)	**<0.001**		0.8780			
**Corsi**								
T0	−0.44(1.10)	−0.86(0.97	0.00	0.00	6.75	0.19	ns	**0.05**
T1	−0.99(1.04)	−0.33(0.97)	0.9633		0.0148			
**Phonological fluency**								
T0	−1.30(0.73)	−1.21(0.77)	0.60	0.2	0.02	0.00	ns	Ns
T1	−1.21(0.82)	−1.08(0.70)	0.5014		0.8780			
**Semantic fluency**								
T0	−1.53(0.84)	−1.51(0.59)	3.46	0.10	0.47	0.01	ns	0.07
T1	−1.34(0.63)	−1.11(1.13)	0.0931		0.8780			
**TROG2**								
T0	75.19 (11.34)	75.94 (15.37)	23.47	0.42	0.32	0.01	**0.01**	**<0.001**
T1	84.63 (9.84)	87.89 (16.58)	**0.0000**		0.8780			

*SST, Standard Speech Treatment; MCNT, Movement Cognition and Narration of the emotions Treatment; η^2^, effect size; ST-Verb Memory, Short Term Verbal Memory; LT-Verb Memory, Long Term Verbal Memory; MBT, Modified Bells Test; TOL, Tower of London; TROG2, Test for Reception Of Grammar (data in standard score); SD, standard deviation; ns, not significant; FDR, false discovery rate correction. Significant results are reported in bold font.*

## Discussion

In this paper, we presented data from an RCT whose aim was to determine the effectiveness of an experimental intervention for the treatment of children with BIF, MCNT, and to compare it to the usual care. Children with BIF are at high risk of school failures and dropouts. For these reasons, an effective intervention able to reduce the occurrence of these events is highly relevant.

Results showed that children in the MCNT group had a significant improvement in their intellectual functioning while children in the SST group did not. This datum is the principal finding of this study, and it is likely due to the improvement in the performance skills that are strictly related to fluid intelligence, which is the capacity to reason in a creative way and to cope with new situations. Moreover, the verbal component of the IQ, associated with crystallized intelligence, showed only a trend toward significance. These results are in agreement with the type of approach used in the MCNT that gave priority to reasoning and planning abilities skills and was less focused on academic knowledge. In particular, the experimental treatment group received intensive training of cognitive abilities with special attention to metacognitive strategies, brainstorming techniques, and elicitation of semantic associations to make conceptual links and improve long term memory. Several studies have investigated the possibility to increase fluid intelligence with targeted cognitive training. The results of these studies have provided controversial results. A metanalysis on the topic showed effective changes in cognitive skills in adults (Au et al., 2015), while another claimed that working memory training produced only short-term effects that do not generalize to tasks remote from the trained ability (Melby-Lervag et al., 2016). The increment in the IQ scores observed in the present study cannot be attributed to any of these considerations since we did not use targeted cognitive training, but a metacognitive approach. Moreover, several pieces of evidence suggest that during development the role of the environment can be crucial, especially for children growing in adverse social environments (Masten and Coatsworth, 1998; Repetti et al., 2002). Our data seem to support this evidence and underlie the importance of intervening with effective approaches during childhood.

Moreover, the MCNT treatment included a set of motivational strategies, such as explicitly underlying the importance of the effort and practice in the increment of their abilities to support their self-efficacy. This approach promoted the motivational systems through the explicitness that intelligence is not a fixed entity but a malleable quality that for any given individual can always be further developed. The individual’s motivation toward achievement is shaped by its implicit theory of intelligence: conceiving of one’s intelligence as a fixed entity is associated with a maladaptive tendency to perform actions to appear capable and avoid negative judgments, whereas conceiving of intelligence as a malleable quality is associated with a more adaptive attitude toward the learning goal of developing that quality (Blackwell et al., 2007).

Another peculiarity of the MCNT treatment was the promotion of cooperative learning, according to Vygotsky’s idea of the importance of learning through communication and interactions with others (Doolittle, 1997). In the MCNT intervention one of the main objectives for the group setting was the involvement of all the children in the group’s activities. To favor positive interdependence, each member was encouraged to participate in the activities according to his/her own strengths and children could seek for the help of the others. The group as a whole was responsible for the achievement of specific goals. This approach has been proven useful to promote positive collaboration and social interactions with greater academic achievements compared to individualistic learning (Johnson et al., 1990).

Unfortunately, the complexity and interdependency of the many factors involved in the MCNT treatment makes it difficult to determine which aspect was most efficacious in ameliorating the BIF condition.

In terms of motor skills, the results of this study showed no improvement for either group. A possible explanation was the use of exergaming devices that probably did not allow for optimal training of the fine motor skills. These data indicate the necessity to reconsider the activities of the Movement Lab.

According to the Psychodynamic Diagnostic Manual-2 (Lingiardi and McWilliams, 2015), all aspects of the mental functioning of the child (among which the capacity for regulation, attention, and learning; the capacity for relationships and intimacy; and the capacity for affective experience, expression, and communication are included) are relevant for the development of the personality. According to this perspective, MCNT was focused on multiple domains and the improvement of children’s emotional and relational competences was one of the main goals of the intervention. Our results demonstrated that the MCNT group improved significantly in terms of socialization abilities and behavior. Conversely, the SST group not only did not improve in either scales and but it also showed a worsening in the CBCL. It should be noted that children belonging to the SST group, at baseline, showed a higher score in terms of Socialization compared to the MCNT group. For this reason, we cannot rule out a ceiling effect in this group. Nevertheless, the data show significant improvement in the socialization skills and behavior in the experimental group, and this is highly relevant due to the importance of these abilities for academic achievements. In the experimental intervention, the Emotion lab was aimed at improving the relational skills of children by means of a better comprehension and narration of their own emotions in everyday experiences. Children were “trained” and helped to increase their emotional competence through a therapeutic intervention centered on the possibility to attribute an emotional meaning to experiences. Behavioral problems in children are often due to the inability to cope with very disturbing emotions and sensations that are not fully understood. Emotional competence is indeed inversely related to several anxiety-related disorders (Mathews et al., 2016). The idea was that taking care of the emotional–relational aspects of BIF children and working toward the improvement of these skills might represent a protective factor against the risk of school failures and the developing of psychopathology in the later stage of life. Our results are thus in line with several studies showing the value of mental state talk, mentalization, and symbolic play in emotional understanding, affect regulation, symptom remission and decrease in disruptive behavior, all relevant elements for the clinical population considered in this study (Halfon et al., 2017, 2019; Gatta et al., 2019; Halfon and Bulut, 2019; Prout et al., 2019a, b).

Moreover, regarding the importance of the intensity of the treatment, two recent studies reported on the efficacy of two intensive interventions for young adults with BIF in the Netherlands: the Assertive Community Treatment (ACT) and the Flexible ACT (Neijmeijer et al., 2018, 2019). These treatments consisted of a wide range of supportive interventions such as psychological treatment, emotion regulation, somatic care, support regarding living, etc. Data showed the efficacy of these interventions in a longitudinal period of 5 years during which patients had significant improvement in social and psychological functioning, in association with a decrement in the number of admissions to mental health care, number of contacts with police and justice, and number of behavioral disorders, with a persistence of the financial and employment problems. Furthermore, a pilot study on a cognitive–behavioral group training for social abilities for adolescents with BIF showed interesting positive results for social competences, and social problem solving, with negative results on related cognitive domains (Nestler and Goldbeck, 2011). Data from these studies, in our opinion, support the idea that a multi-domain approach that also includes training of cognitive abilities is necessary for this vulnerable population.

Finally, changes in specific cognitive abilities were observed in both groups. The lack of the no-treatment group does not allow us to make a final inference about these data because factors other than treatment, such as maturation and test-learning effects, could be involved. Nevertheless, since all tests used were corrected for age and due to the long interval between pre and post-treatment evaluations, we considered it plausible that data reflected the effects of both treatments. In particular, children in the MCNT group improved in tasks exploring selective attention, visual-spatial short-term memory, verbal long- and short-term memory, verbal comprehension and executive planning. These findings likely reflect the type of work that was done in the cognitive lab, which was centered on cognitive flexibility, memorization strategies, problem solving, verbal comprehension, planning and executive functions. The SST group, working on learning abilities and transversal aspects such as attention, memory and verbal comprehension, also showed improvement in sustained selective attention, verbal short- and long-term memory as well as verbal comprehension and executive planning. Due to the broad influence that verbal comprehension has on virtually all cognitive abilities, both treatments trained children in this aspect. In the SST group, improved cognitive abilities were not coupled with changes in their adaptive/behavioral skills. Although our study involved only a small sample of subjects, this datum suggests that focusing only on the cognitive performance in this population is not sufficient to prevent behavioral, social and mental problems. Due to the high level of stress and adversities that children with BIF face in their school, family and social life, they suffer a much greater risk in developing a problematic personality profile with the consequent risk of psychopathology, highlighting the need to be properly supported in their emotional/relational needs.

The present study has some major limitations. The first relates to the lack of a no-treatment group, which prevents us from distinguishing between treatment effects and potential biases related to children maturation or learning effects. The second limitation concerns the different intensity between treatments. It is possible that some of the changes observed were due to this bias. Considering the precise domains in which the improvements occurred in each group, and the worsening observed in the behavior of the SST group, it is unlikely that treatment intensity can explain all the changes that we observed. In particular, children in the SST group, despite the lower intensity of the treatment, did show improvement in all the abilities that were trained such as verbal memory, verbal comprehension, selective attention, and executive planning, but no improvements were observed for visual-spatial memory, IQ and behavior that were not trained. These results are not conclusive evidence but they do suggest that intensity of training alone cannot explain all the differences observed.

Another significant limitation is represented by the small number of participants that prevents generalization of the present data to the whole population of children with BIF. Larger scale studies will be necessary to further explore the efficacy of the MCNT approach in the treatment of children with BIF, also in the long term.

## Conclusion

Considering the poor prognosis of children with BIF in the long term, with educational and vocational failures and the risk to develop psychopathology, we consider our data highly relevant as they demonstrate the possibility to improve competences at multiple levels with an intensive and integrated training. Although additional studies with a long-term follow up will be necessary, we hypothesize that the improvements obtained after MCNT might represent a protective factor able to reduce the risk of poor outcome. Indeed, improving the fluid intelligence and the emotional/behavioral competencies is likely to enhance the ability of children with BIF to cope with their everyday challenges in school, family and social contexts, promoting resilience (Goldman et al., 2016). The results of the present study indicate the opportunity to implement multimodal, intensive and timely rehabilitation interventions in children with BIF. Cost-efficacy analyses will be necessary to determine the feasibility to incorporate this approach within the healthcare provided by the national health system. These analyses should also consider the high risk of children with BIF to develop mental and physical problems, and poverty.

## Data Availability Statement

The datasets used and analyzed during the current study are available from the corresponding author on reasonable request.

## Ethics Statement

The studies involving human participants were reviewed and approved by the Ethics Committee of the IRCCS Fondazione Don Carlo Gnocchi Onlus, and the Ethics Committee of the ASST S. Paolo and S. Carlo Hospital. Written informed consent to participate in this study was provided by the participants’ legal guardian/next of kin.

## Author Contributions

MZ, FB, MC, and VB conceived the study and wrote the manuscript. GB, VB, AG, MW, and MZ executed the study and MPC helped with implementation. SD helped with statistical analyses. All authors contributed to refinement of the manuscript and approved the final content.

## Conflict of Interest

The authors declare that the research was conducted in the absence of any commercial or financial relationships that could be construed as a potential conflict of interest.

## Footnotes

1 http://graphpad.com/quickcalcs/randMenu/

2 https://www.erickson.it/it/
